# Digital Self-Management in Support of Patients Living With Chronic Pain: Feasibility Pilot Study

**DOI:** 10.2196/23893

**Published:** 2020-10-23

**Authors:** Katrine Bostrøm, Elin Børøsund, Cecilie Varsi, Hilde Eide, Elise Flakk Nordang, Karlein MG Schreurs, Lori B Waxenberg, Karen E Weiss, Eleshia J Morrison, Milada Cvancarova Småstuen, Audun Stubhaug, Lise Solberg Nes

**Affiliations:** 1 Department of Digital Health Research Division of Medicine Oslo University Hospital Oslo Norway; 2 Institute of Clinical Medicine Faculty of Medicine University of Oslo Oslo Norway; 3 Science Centre Health and Technology University of South-Eastern Norway Drammen Norway; 4 Department of Psychology, Health & Technology University of Twente Enschede Netherlands; 5 Department of Clinical and Health Psychology University of Florida Gainesville, FL United States; 6 Department of Anesthesiology and Pain Medicine University of Washington School of Medicine Seattle, WA United States; 7 Department of Psychiatry and Psychology College of Medicine and Science Mayo Clinic Rochester, MN United States; 8 Faculty of Health Sciences Oslo Metropolitan University Oslo Norway; 9 Department of Pain Management and Research Oslo University Hospital Oslo Norway; 10 Regional Advisory Unit on Pain Oslo University Hospital Oslo Norway

**Keywords:** chronic pain, feasibility, acceptability, self-management, eHealth, digital, cognitive-behavioral pain, usability, user centered

## Abstract

**Background:**

Chronic pain can be complex and taxing to live with, and treatment and support require a multicomponent approach, which may not always be offered or available. Smartphones, tablets, and personal computers are already incorporated into patients’ daily lives, and therefore, they can be used to communicate, educate, and support self-management. Although some web-based self-management interventions exist, research examining the evidence and effect of digital solutions supporting self-management for patients living with chronic pain is limited, findings are inconclusive, and new innovative ideas and solutions are needed.

**Objective:**

This feasibility pilot study aimed to explore the system use, perceived usefulness, ease of use, and preliminary effects of EPIO, an app-based cognitive-behavioral pain self-management intervention program for patients living with chronic pain.

**Methods:**

The EPIO intervention was delivered in a blended-care model containing (1) one face-to-face introduction session, (2) nine cognitive behavior–based pain self-management modules, delivered in an app-based format for smartphones or tablets, and (3) one follow-up phone call at 2 to 3 weeks after the introduction session. Patients living with chronic pain (N=50) completed pre-post outcome measures at baseline and 3 months after the introduction session, with registration of system use (ie, log data) until 6 months. The use, perceived usefulness, and ease of use of the EPIO program were examined through system use data, as well as a study-specific use/usability questionnaire and the System Usability Scale (SUS). Outcome measures to test feasibility of use and estimate preliminary effects included the Brief Pain Inventory, health-related quality of life (HRQoL) scale, Hospital Anxiety and Depression Scale, Self-Regulatory Fatigue scale, Pain Catastrophizing Scale, and Chronic Pain Acceptance Questionnaire.

**Results:**

Participants (N=50) had a median age of 52 years (range 29-74 years) at inclusion and were mainly female (40/50, 80%). Thirty-one participants completed at least six of the nine modules within the 3-month study period (62% completion rate). Forty-five participants completed outcome measures at 3 months, and the EPIO program was rated as useful (ie, “totally agree” or “agree”; 39/45, 87%) and easy to use (42/45, 93%), and as having easily understandable exercises (44/45, 98%). The average overall system usability (SUS) score was 85.7, indicating grade A and excellent system usability. Preliminary psychosocial outcome measure estimates showed primarily nonsignificant pre-post intervention improvements at 3 months, but with significant positive effects related to some aspects of HRQoL (bodily pain, *P*=.02 and change, *P*=.049).

**Conclusions:**

Digital self-management intervention programs may be of use and support for patients living with chronic pain. In this feasibility study, EPIO showed an acceptable program completion rate and was rated as useful and easy to use, with excellent user satisfaction. Program optimization and efficacy testing in a large-scale randomized controlled trial are warranted and in progress.

**Trial Registration:**

ClinicalTrials.gov NCT03705104; https://clinicaltrials.gov/ct2/show/NCT03705104

## Introduction

### Background

Chronic pain is complex and taxing to live with, and how patients perceive and relate to pain is based on an interplay of biomedical, psychosocial, behavioral, and cultural factors [1]. Given this intricacy, chronic pain is optimally managed by treatments that address not only biological factors [2,3], but also psychological and social influences and consequences. The complexity, demands, and challenges of living with chronic pain may lead to a draining of capacity to self-regulate [4-6]. Helping patients to build or strengthen their self-regulatory capacity and support motivation to engage in pain self-management strategies can, therefore, be important [7]. International clinical guidelines also recommend the inclusion of self-management interventions in routine treatment for patients living with chronic pain [2].

Psychosocial interventions based on cognitive behavioral therapy (CBT) [8] and/or acceptance and commitment therapy (ACT) [9], aiming to support coping and self-management for patients living with chronic pain [10,11], have been shown to be associated with improved quality of life, pain acceptance, functioning, and self-efficacy, as well as reduced pain, anxiety, and depressive symptomatology [12-14]. Unfortunately, such individual or group in-person psychosocial interventions are not always an option for patients living with chronic pain [15]. Possible barriers include lack of accessibility of services, personal preferences, the medical condition itself, lack of insurance coverage, and geographical distance [16,17]. Given the limited availability and options of in-person psychosocial interventions for patients living with chronic pain, new and innovative ideas and solutions are needed [18].

Digital solutions in the form of eHealth solutions, defined as the use of digital communication-based technology to provide health care and support self-management of health conditions [19], may provide innovative options for patients living with chronic pain [20]. Patients with chronic pain have also reported being interested in eHealth interventions in support of self-management [7,21], and existing eHealth interventions for self-management of chronic pain have shown promise in terms of the potential to address unmet needs, support psychological well-being, strengthen self-efficacy, and increase flexibility [18,22]. However, findings and indications of efficacy for such pain-related interventions are still limited and mixed.

Most patient-oriented apps for people living with chronic pain only provide information about pain or about the illness, including ways to check symptoms and track medication use [23]. Few eHealth pain-related apps provide information about coping and self-management strategies [24,25], and even though some web-based CBT or ACT-based interventions have been tested in support of people living with chronic pain, findings are still inconclusive and interventions need further testing, also in app format [13,22,26-28]. Systematic reviews have concluded that eHealth interventions are more likely to be successful if developed with a user-centered focus, increasing the likelihood of matching the user’s needs and requirements [29,30]. However, systematic literature reviews examining the development and use of pain-related eHealth apps indicate that very few of these programs are developed with the involvement of health-care professionals and actual end users (ie, patients with chronic pain), and only a few existing pain-related apps appear to be based on a theoretical and evidence-based rationale [31,32]. These aspects emerge as major limitations of eHealth pain self-management interventions so far. In addition, few existing studies report system use and level of engagement (ie, app activity), and/or satisfaction/usability with eHealth pain self-management programs to date [33]. Given these challenges identified by existing scientific literature, new extensive research and innovative solutions are required to show the feasibility, usefulness/usability, and effectiveness of eHealth interventions supporting self-management for patients living with chronic pain [20,34].

In response to existing research and recommendations, the current research team has examined users’ (ie, patients living with chronic pain) [21] and health care providers’ [35] inputs related to needs and requirements for a potential eHealth pain self-management intervention [21,35]. Incorporating findings [21,35] and combining these with existing clinical and research evidence for the effectiveness of CBT/ACT-type interventions [36-39], the team subsequently designed and developed a cognitive-behavioral pain self-management eHealth intervention called *EPIO* (inspired by the Greek goddess for the soothing of pain, Epione), aiming to support patients living with chronic pain (ie, chronic pain in general, not pain/pain condition specific) [21,26,35]. This study builds on this research line.

### Objectives

To enable the effective evaluation of complex interventions, the Medical Research Council recommends initial intervention testing and refinement to ensure intervention feasibility [40]. This feasibility pilot study therefore aimed to assess system use (ie, user app activity), perceived usefulness, and ease of use of the EPIO intervention program in order to identify needs for adjustments and to enable optimization in preparation for a future randomized controlled trial (RCT). The current feasibility pilot also aimed to explore preliminary efficacy findings (ie, pain interference, health-related quality of life [HRQoL], anxiety and depression, self-regulatory fatigue, pain catastrophizing, and pain acceptance) using unadjusted exploratory pre-post intervention analyses.

## Methods

### Description of the EPIO Intervention Program

The *EPIO* intervention program was designed and developed by a collaborative research team, consisting of scientists, health care providers, eHealth experts, content and system developers, and end-user representatives (ie, patients living with chronic pain) [26]. Content development [26] was based on well-known evidence-based aspects from CBT, with some integrated aspects of ACT, focusing on self-management and coping for patients living with chronic pain in general [7,11,15,30,41,42]. Focusing on chronic pain in general, the EPIO intervention program is so far not developed to be pain type/pain condition specific. The EPIO program contains nine modules designed with several interconnected parts of information and education (eg, pain physiology, coping strategies, thought challenges, and the importance of activity balance) and a variety of self-management–based exercises for patients living with chronic pain (eg, diaphragmatic breathing, graded behavioral activation, mindfulness, and progressive muscle relaxation) [26,41,43]. The nine modules in the EPIO program include the following topics: (1) information about pain, (2) balance, (3) thoughts and feelings, (4) stress and coping, (5) what is important to me (ie, values), (6) behaviors and lifestyle, (7) communication, relations, and social support, (8) coping during difficult times, and (9) summary and the road ahead [26].

To encourage program content practice, each module has to be open for 3 days (ie, practicing mode) before the next module will open. To provide structure and to allow individualization, the first five EPIO modules are sequential, while the order of modules 6 to 8 can be chosen. In addition, participants can create their own favorite list by highlighting exercises and can receive reminders according to their own needs. Participants can also choose between reading and listening to the program at any time. To ensure availability, the program can also be used while participants are offline. Details for the design, development, and content of EPIO are presented elsewhere [26]. The EPIO intervention is delivered in a blended-care model containing the following: (1) one face-to-face introduction session; (2) nine primarily CBT-based pain self-management modules [26], delivered in an app-based format for smartphones or tablets; and (3) one follow-up phone call conducted at 2 to 3 weeks after the introduction session.

### Study Design

A pre-post intervention study without a control group was employed in this study, with all participants receiving the EPIO intervention. Outcome measures to test feasibility of use and derive estimates of preliminary efficacy were collected at baseline and at 3 months after the introduction session. In addition, data of system use (ie, log data) were collected for 6 months, and extracted at 3 and 6 months after the introduction session. Feasibility conceptualization was guided by Bowen et al [44] exploring (1) *acceptability* (to what extent is the EPIO program judged as suitable, satisfying, or attractive to program recipients); (2) *demand* (to what extent is EPIO likely to be used, ie, exploration of the actual use of the program); and (3) *limited efficacy testing* (does the EPIO program show promise of being successful with the intended population?).

### Participants and Recruitment

Information about the study was communicated through the research project website [45], through the initiating institution (Oslo University Hospital), and through collaborating partners, including local health care services and primary care practices. Study and recruitment information was also advertised through social media channels and through patient organizations’ web pages. The inclusion criteria were as follows: age ≥18 years, living with chronic pain in general (ie, not pain/pain condition specific), pain duration ≥3 months (self-reported), access to a smartphone or tablet, being able to understand oral and written Norwegian, and being able to attend an in-person introduction session. The exclusion criteria were as follows: having untreated severe mental illness, migraine, or cancer-related pain (all self-reported). Participants were recruited between January and May 2019.

### Study Procedure

The study was approved by the Regional Committee for Medical and Health Research Ethics (REK 2018/8911) and the Oslo University Hospital Institutional Review Board equivalent function (PVO 2017/6697). All participants provided written informed consent. Participants attended one in-person introduction session where they were introduced to the self-management program in the introduction session and received help downloading the EPIO app from the App Store or Google Play Store, with instructions on how to get started. They also received a follow-up phone call from the study staff 2 to 3 weeks after the introduction session to see how things were going and whether there were any questions. If the participants had any additional questions or feedback (eg, technical issues) related to the EPIO program during the study, they could contact the study staff by phone. System use was logged for 6 months through a secure server at Services for Sensitive Data (TSD; University of Oslo). Outcome measures were completed through a secure TSD server online at baseline (ie, before the introduction session) and at 3 months after the introduction session (ie, at a 3-month follow-up). Program completers were defined as participants completing at least six out of the nine modules (67%) of the EPIO program in the 3-month study period [46].

### System Use

For study purposes, participants were encouraged to try to complete all nine app-based modules within 3 months, but they could continue to use the program for as long as they wanted. System use (ie, user app activity) and program progress were automatically logged for 6 months through a secure server (ie, TSD) using an encrypted connection. To explore the extent to which the EPIO program was used by participants (ie, *demand*) [44], system use log data (ie, app activity) were extracted at 3 and 6 months after the introduction session.

### Perceived Usefulness and Ease of Use

To explore the extent to which the EPIO program was judged as suitable, satisfying, or attractive to intervention recipients (ie, *acceptability*) [44], participants completed a six-item study-specific questionnaire (Multimedia Appendix 1), as well as the System Usability Scale (SUS) [47] at the 3-month follow-up. The study-specific questionnaire was based on previous experience with developing eHealth apps in the research team [26,48] and was guided by the Technology Acceptance Model (TAM) [49]. The first three items in the questionnaire, inspired by Davis [50], measure participant program perception as follows: (1) the program was easy to use, (2) the exercises were easy to understand, and (3) the program was useful. Response options range from *totally agree* to *totally disagree*. The remaining items in the study-specific questionnaire are open-ended questions designed to gather information related to participants’ perceived usefulness and ease of use as follows: (4) What did you like the best? (5) What did you like the least? and (6) What are your suggestions for improvement? The SUS [47] is a 10-item questionnaire with five response options ranging from *strongly disagree* to *strongly agree*. SUS scoring yields a single number representing a composite measure of the overall usability of the system being studied. Scores are to be summarized and multiplied by 2.5, leading to a value range of 0 to 100, and 68 is considered the average score. A score above 80.3 can be interpreted as grade A (ie, the top 10% of scores), which equals excellent system usability [51].

### Preliminary Effects: Outcome Measures

Outcome measures were collected at baseline (ie, before the introduction session) and at the 3-month follow-up*.* At baseline, patients also completed a study-specific demographic and disease-related measure. To test feasibility of use, explore preliminary effects, and assess whether the EPIO program showed promise of being successful in the intended patient population (ie, *limited efficacy testing*), participants completed several psychosocial outcome measures.

*Pain interference* was measured with seven items from the short form of the Brief Pain Inventory (BPI) [52], a measure of the impact of pain on daily function. The BPI has acceptable internal consistency and reliability and has been validated in a Norwegian chronic pain population sample [53]. The score range of BPI is 0 to 10, with higher scores indicating higher pain interference.

*HRQoL* was measured with the noncommercial SF-36 Short Form Health Survey (RAND-36) [54,55], a 36-item measure of physical, role, emotional, cognitive, and social function, as well as physical, general, and global health. The RAND-36 has acceptable internal consistency and reliability [54] and has been validated in a Norwegian population sample with chronic pain [55]. The score range of RAND-36 is 0 to 100, with higher subscale scores indicating better HRQoL.

*Anxiety and depressive* symptoms were measured with the Hospital Anxiety and Depression Scale (HADS) [56], a 14-item measure of anxiety and depressive symptomatology, validated as a unidimensional measure of psychological distress. The HADS has acceptable internal consistency and reliability [56]. The score range of HADS is 0 to 21 for both scales, with higher scores indicating a higher presence of anxiety or depression.

*Self-regulatory fatigue* was measured with the Self-Regulatory Fatigue 18 (SRF-18) [57], an 18-item scale measuring self-regulatory capacity with cognitive, emotional, and behavioral components. The SRF-18 has acceptable internal consistency and reliability [57]. The score range of SRF-18 is 18 to 90, with higher scores indicating higher self-regulatory fatigue.

*Pain catastrophizing* was measured with the Pain Catastrophizing Scale (PCS) [58,59], a 13-item scale measuring catastrophic thinking and maladaptive responses to pain. Three subscales measure helplessness, magnification, and rumination. The PCS has acceptable internal consistency and reliability, and has been validated in a Norwegian population sample with chronic pain [59]. The score range of the PCS is 0 to 52, with higher scores indicating higher catastrophic thoughts and feelings about pain.

*Pain acceptance* was measured with the short form of the Chronic Pain Acceptance Questionnaire (CPAQ-8) [60,61], an eight-item scale measuring pain acceptance. The CPAQ-8 has acceptable internal consistency and reliability [61], and has been validated in a Norwegian population sample with chronic pain [60]. The score range of the CPAQ-8 is 0 to 24, with higher scores indicating a higher acceptance of pain.

### Qualitative Analyses

Qualitative data from the open-ended questions in the study-specific questionnaire were analyzed using an Excel spreadsheet, according to a thematic analysis process (ie, coding reliability) as described by Braun and Clark [62]. The first author (KB) performed the analyses of the data, in collaboration with a coauthor (EB). The data were grouped as domains, directly guided by the study questions (ie, what did you like the best, what did you like the least, and suggestions for improvement), before categories were derived.

### Statistical Analyses

Data were analyzed using the Statistical Program for Social Sciences (SPSS), version 25 (IBM Corp). Data on baseline characteristics, system log data, and usefulness/ease of use data are presented as medians and ranges for continuous variables and as proportions with percentages for categorical variables. Paired samples *t* tests were used to assess possible pre-post intervention changes. To explore potential group differences in outcome measures, demographics, and program progress, univariate linear regression analyses were conducted. All statistical tests were two-sided. *P* values <.05 were considered statistically significant. As this was a feasibility pilot study, results were considered exploratory and no correction for multiple testing was performed [63].

## Results

### Recruitment, Participant Flow, and Sample Description

**Figure 1:**
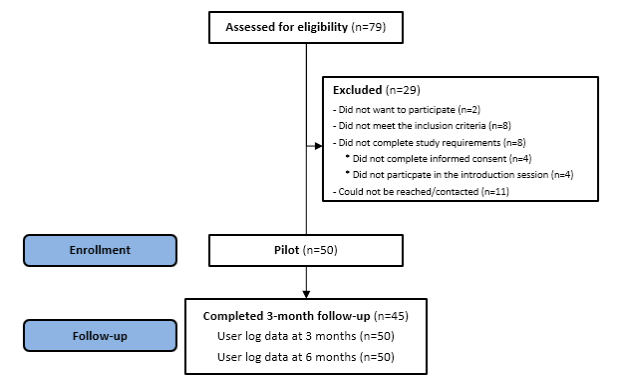
Recruitment and participant flow.

Between January and May 2019, 79 patients living with chronic pain were referred for potential study participation. Of these, 29 were excluded (ie, did not meet the inclusion criteria, did not complete study requirements, could not be reached, or declined to participate). Fifty patients with chronic pain were included in the study and received the EPIO intervention. Forty-five of the initial participants completed the 3-month outcome measures. Of the five participants who did not complete the 3-month outcome measures, two described a worsened health condition as the reason. No other reasons were provided. Apart from age (ie, noncompleters were significantly older than completers, 62.2 years versus 51.3 years, *P*=.02), there were no differences between noncompleters and completers with regard to baseline variables. Figure 1 provides a summary of the recruitment and participant flow.

Participants (N=50) were primarily Caucasian (48/50, 96%), had a median age of 52 years (range 29-74 years) at inclusion, and were mainly female (40/50, 80%). Forty-one participants provided self-reported details related to their pain conditions, including pain related to unspecific musculoskeletal pain (eg, back and neck pain [16/41, 39%], unspecified disc disorder [8/41, 19%], osteoarthritis [7/41, 17%], fibromyalgia [7/41, 17%], neuropathy [7/41, 17%], complex regional pain syndrome [4/41, 10%], injuries [5/41, 12%], and surgeries [3/41, 7%]), with more than half of the participants reporting more than one diagnosis (23/41, 56%). The majority (37/50, 74%) of the participants reported having lived with pain for 5 years or longer. Table 1 presents the baseline demographics and illness characteristics of the participants.

**Table 1:** Baseline demographics and illness characteristics (N=50).

Variable		Value, n (%)
**Gender**		
	Female	40 (80)
	Male	10 (20)
**Marital status**		
	Married/cohabitating	29 (58)
	Single/divorced	21 (42)
**Education**		
	Elementary/high school	17 (34)
	University/college ­<4 years	21 (42)
	University/college ­≥4 years	12 (24)
**Employment**		
	Full-time/part-time work	14 (28)
	Sick leave/disability benefits	29 (58)
	Retired/others	7 (14)
**Years living with pain**		
	1-3 years	10 (20)
	3-5 years	3 (6)
	5-10 years	13 (26)
	>10 years	24 (48)
**Health services usage^a^**		
	General practitioner	47 (94)
	Physiotherapy/physical therapy	40 (80)
	Psychology	16 (32)
	Pain physician/pain specialist services	6 (12)
	Pain clinic	16 (32)
	Occupational therapy	3 (6)
	Rehabilitation	16 (32)
	Healthy life centers	3 (6)
	Educational courses	9 (18)
	Other	11 (22)

^a^Participants could report having received several types of health services during the course of their illness.

### System Use

**Figure 2:**
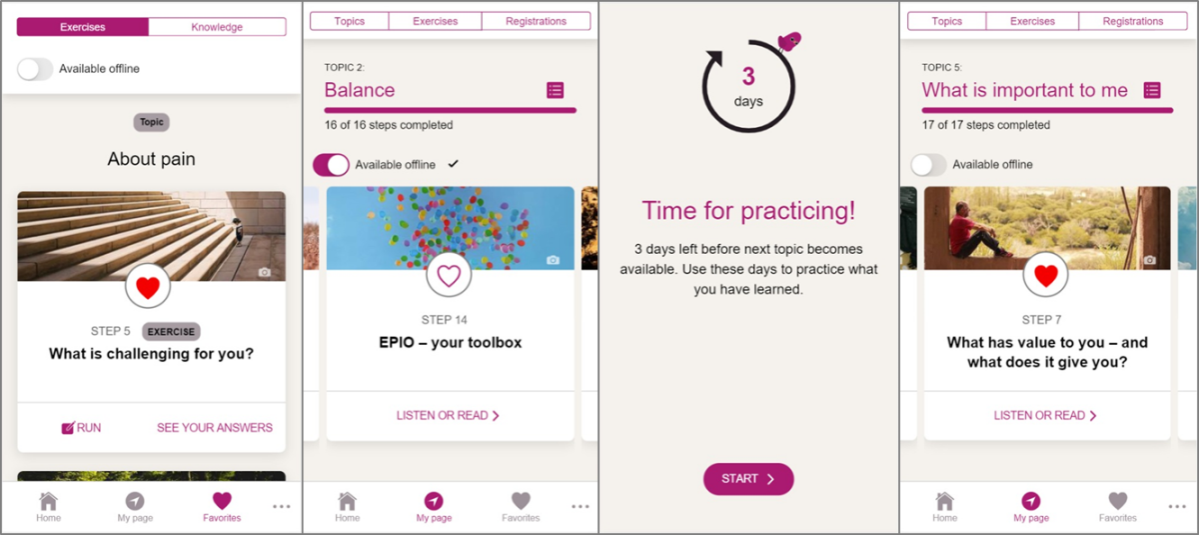
Example screenshots from the EPIO intervention program. From the left: (1) Exercise example about daily challenges; (2) Module about activity pacing; (3) Practicing mode; and (4) Module about values.

Thirty-one participants completed at least six of the nine EPIO program modules within the 3-month study period, yielding a 62% intervention completion rate at 3 months. Fourteen (28%) participants completed all nine modules within 3 months. Noncompleters (ie, completing less than six modules in the 3-month study period) completed an average of two modules in 3 months. Of the EPIO intervention program exercises, the top three exercises repeated most frequently during the 3-month study period were as follows: “What is challenging to you,” “Choice of fun activities,” and ”What kind of thoughts do you use.” For example screenshots of the EPIO program, refer to Figure 2.

While the pre-post intervention study period was 3 months for practical purposes, system use was monitored for 6 months. At the 6-month follow-up, 32 (64%) participants had completed at least six out of the nine program modules. Nineteen (38%) participants had completed all nine modules at the 6-month follow-up.

### Perceived Usefulness and Ease of Use

At the 3-month follow-up, 45 participants (90%) completed measures related to their satisfaction with the EPIO program. In the study-specific use/usability questionnaire, the EPIO program was rated as useful (ie, *totally agree* or *agree*; 39/45, 87%) and easy to use (42/45, 93%), and as having easily understandable exercises (44/45, 98%). Main findings from the open-ended questions showed that a majority of the participants reported appreciating the exercises (eg, the relaxation and diaphragmatic breathing exercises), the combination of exercises and educational information, the easy access, and the functionality of being able to choose between reading and listening or being able to do both. The mean system usability (ie, SUS) score was 85.7 (SD 12.9), indicating grade A, which equals excellent (ie, score >80.3) system usability. Even though few men participated in this study, there were some indications of gender differences, with more women (85%) than men (50%) rating the program as useful. Additionally, there were some differences related to education, with higher educated participants (ie, >4 years of university) having significantly higher SUS scores than lower educated participants (ie, elementary/high school, *P*=.04).

### Preliminary Effects: Pre-Post Interventions Results

Preliminary pre-post intervention findings at the 3-month postintroduction session did not reach statistical significance for the majority of the psychosocial outcome measures (details are provided in Table 2). HRQoL findings indicated statistically significant improvements from baseline to postintervention for “bodily pain” (mean difference [MD] 5.1; *P*=.02) and the single item “change” (ie, perceived change in health) (MD 5.6; *P*=.049), and there was a trend toward significant improvements for the Role Physical scale, but the result was not statistically significant (MD 10.0; *P*=.07). There was a high degree of heterogeneity in the data, reflected in large values of variance and subsequently broad CIs for the point estimates in a number of subscales (eg, HRQoL Role Emotional with a 10-point positive score change, but with CI −3.3 to 24.0 and consequently no statistical significance, *P*=.13). There was also a trend toward significant reduced pain catastrophizing, but the result was not statistically significant (MD 1.8; *P*=.06). Scores related to anxiety, depression, and self-regulatory fatigue remained stable. Moreover, the results after 3 months indicated statistically significant lower pain acceptance (“Willingness to accept” subscale, MD 0.9; *P*=.03; total score, MD −1.4; *P*=.02).

**Table 2:** Preliminary effects: pre-post intervention changes in psychosocial outcomes (n=45).

Psychosocial outcomes			Baseline, mean (SD)		3-month follow-up, mean (SD)		Mean difference (95% CI)		*P* value	
Pain interference (BPI^a^)^b^			4.5 (2.1)		4.8 (2.1)		0.3 (−0.3 to 0.8)		.31	
**HRQoL^c^** **(RAND-36^d^)**										
	Physical function	60.9 (24.6)		61.3 (25.4)		0.4 (−3.1 to 3.9)		.80		
	Role physical	17.2 (31.5)		27.2 (36.5)		10.0 (−0.9 to 20.9)		.07		
	Bodily pain	35.3 (13.4)		40.4 (15.9)		5.1 (0.7 to 9.5)		.02		
	General health	46.3 (19.6)		48.9 (19.7)		2.6 (−1.2 to 6.3)		.18		
	Vitality	31.9 (21.0)		34.1 (20.8)		2.2 (−2.4 to 6.8)		.34		
	Social function	50.3 (24.9)		52.8 (24.1)		2.5 (−2.4 to 7.4)		.31		
	Role emotional	56.3 (45.4)		66.7 (40.8)		10.4 (−3.3 to 24.0)		.13		
	Mental health	63.7 (18.1)		65.1 (17.9)		1.3 (−1.2 to 3.8)		.29		
	Change	49.4 (25.3)		55.0 (23.0)		5.6 (0.0 to 11.1)		.049		
Anxiety (HADS-A^e^)			7.9 (3.6)		8.0 (4.0)		0.0 (−0.8 to 0.9)		.96	
Depression (HADS-D^f^)			5.8 (3.2)		5.8 (3.9)		0.1 (−0.6 to 0.6)		>.99	
Self-regulatory fatigue (SRF-18^g^)			53.7 (9.2)		53.3 (9.2)		0.4 (−2.5 to 1.6)		.66	
**Pain catastrophizing (PCS^h^)**										
	Rumination	7.4 (3.9)		6.9 (4.0)		0.6 (−1.3 to 0.2)		.12		
	Magnification	3.3 (2.4)		3.0 (2.2)		0.3 (−0.8 to 0.1)		.14		
	Helplessness	8.4 (4.8)		7.6 (5.2)		0.9 (−1.9 to 0.2)		.11		
	Total score	19.2 (9.9)		17.4 (10.4)		1.8 (−3.7 to 0.1)		.06		
**Chronic pain acceptance (CPAQ^i^)**										
	Willingness	14.0 (2.8)		13.1 (3.1)		0.9 (−1.7 to −0.1)		.03		
	Activity engagement	14.3 (3.3)		13.8 (3.3)		0.5 (−1.4 to 0.4)		.23		
	Total score	28.3 (5.2)		26.9 (5.3)		1.4 (−2.7 to −0.2)		.02		

^a^BPI: Brief Pain Inventory.

^b^Subscale of the Brief Pain Inventory (score range 0-10; a higher score indicates higher interference in life).

^c^HRQoL: health-related quality of life.

^d^RAND-36: RAND 36-Item scale (score range 0-100; a higher score indicates higher emotional well-being).

^e^HADS-A: Hospital Anxiety and Depression Scale-Anxiety subscale (score range 0-21; a higher score indicates a higher degree of anxiety).

^f^HADS-D: Hospital Anxiety and Depression Scale-Depression subscale (score range 0-21; a higher score indicates a higher degree of depression).

^g^SRF-18: Self-regulatory Fatigue 18 scale (score range 18-90; a higher score indicates higher self-regulatory fatigue).

^h^PCS: Pain Catastrophizing Scale (score range 0-52; a higher score indicates higher catastrophizing).

^i^CPAQ: Chronic Pain Acceptance Questionnaire (score range 0-52; a higher score indicates a higher acceptance of pain).

### Program Completion and Relations to Psychosocial Outcomes

The participants who completed the EPIO program (ie, completed six or more modules) within the 3-month study period had higher mean pain interference and lower emotional well-being scores at baseline compared with those who did not complete the program (ie, completed five or less modules). However, these differences were not statistically significant (both *P=*.28).

### Preparation for Optimization and a Randomized Controlled Trial

In order to optimize the EPIO intervention and prepare for efficacy testing in an RCT, system use, perceived usefulness, and ease of use findings from this study were employed to prioritize and address the needs for further adjustments to the EPIO intervention program after the feasibility pilot. For example, as the 3-day delay between modules (ie, practicing mode) received mixed feedback, with some preferring to move forward more rapidly and others liking the practicing mode, modules 1 and 2 were set to open simultaneously as the introduction session and module 1 had overlapping themes. Additionally, based on system log data showing increased program activity right before and right after the 2 to 3 week follow-up phone call, a decision was made to add a second follow-up phone call (eg, at 6-7 weeks) for the future RCT.

## Discussion

### Feasibility of eHealth in Chronic Pain

Feasibility studies play an important role in the planning of RCTs to examine novel interventions or to examine a combination of existing interventions in new patient populations or recruitment settings [44]. Patients with chronic pain have reported being interested in eHealth interventions in support of self-management [7,21]. Given the early stage of evidence-based eHealth interventions, however, the need for more studies reporting on the feasibility, usability, and efficacy of eHealth intervention programs for patients living with chronic pain is evident [20,34]. This feasibility pilot study therefore examined the system use, perceived usefulness, ease of use, and preliminary effects of EPIO, an app-based cognitive-behavioral pain self-management intervention for patients living with chronic pain (ie, chronic pain in general, not pain/pain condition specific).

### Principal Findings

In the 3-month study-period, 62% (31/50) of participants completed at least six out of nine modules of the EPIO program. The participants rated the program as useful (39/45, 87%) and easy to use (42/45, 93%), and mentioned the presence of easily understandable exercises (44/45, 98%). System usability was rated as excellent, and although mainly nonsignificant (details are provided in Table 2), preliminary psychosocial outcome measures indicated some positive impact related to HRQoL. The repeated use of multiple self-management exercises in the EPIO program also suggests that exercise variety may be particularly of interest and may support program use and engagement for patients living with chronic pain. As suggested by scientific literature reviews examining status and limitations of eHealth interventions [20,32,34], the EPIO intervention program was developed based on existing evidence, in close collaboration among scientists, user representatives, and health care providers [26], which may have contributed to the positive feasibility and acceptability, including system use, perceived usefulness, and ease of use findings, noted in this study.

Participants’ engagement is a precondition for the effectiveness of self-management interventions [64]. In this feasibility pilot, participants were therefore encouraged to spend as much time as possible becoming familiar with the EPIO program and to practice the content and variety of exercises as much as possible. The 62% completion rate (ie, completing at least six modules) during the 3-month study period is likely lower than the completion rates for equivalent in-person interventions. However, adherence/completion rates have emerged as a challenge for eHealth interventions, sometimes being as low as 20% to 40%, and the 62% completion rate in this study can therefore be considered acceptable.

System log data examinations also revealed slightly higher program completion rates at the 6-month follow-up than at the 3-month follow-up. Research indicates that the complexity, demands, and challenges of living with chronic pain may lead to a draining of the capacity to self-regulate cognitive, emotional, and behavioral activities, sometimes particularly related to executing functioning [4-6]. If patients living with chronic pain struggle with the many important decisions and the behavioral changes often required for successful self-management, finding new ways to support patients with chronic pain to maintain engagement and adhere to self-management programs may be important [65]. Given the findings from this study, it is possible that patients living with chronic pain could benefit from having more time (ie, >3 months) to process the program information than initially estimated by this research team. Participants in this study were however given access to the EPIO program for as long as they wanted. Therefore, despite the limited increase in use and completion rates from 3 to 6 months, the apparent continued use of the EPIO program in the poststudy period (ie, >3 months) may indicate that participants took their time engaging in the EPIO intervention program and found the program useful after study completion.

Program completers in this study reported higher pain interference and lower emotional well-being at baseline compared with noncompleters. This could potentially suggest that higher levels of pain interference and lower emotional well-being may be positively associated with program interest, motivation for change, engagement, and completion. Such indications could be of great interest for the development and use of future eHealth interventions. However, in this feasibility pilot study, the associations among program completion, progress, and psychosocial outcomes were nonsignificant (*P=*.28). Conclusions cannot be made, and this issue needs to be further explored by future research in RCTs with larger study populations.

Despite some indications that older participants may have been more likely to be noncompleters in this study, only five individuals were study noncompleters by 3 months, and conclusions cannot be made. There are however some suggestions that high age could be associated with lower eHealth use, with younger people expressing more willingness and interest in using eHealth and older adults expressing worry about losing personal contact with their physicians if they start to use eHealth [66]. Research nevertheless also suggests that older adults seem to adhere to eHealth technology longer than younger people after starting to use such technology [66]. The link between age and eHealth use is not clear, as several studies have failed to show any difference in eHealth use based on age, and more research in this area is needed [66].

The outcomes of eHealth self-management interventions seem to depend on patients’ motivation and adherence [7,25,64,67]. The adherence issues related to eHealth interventions are of great concern and need to be addressed in order for end users to achieve the intended intervention benefit. There are some indications that eHealth interventions may yield better adherence and subsequent effects when combined with face-to-face/in-person support [64]. The EPIO intervention was delivered in a blended-care model, which aimed to increase the motivation and likelihood of acceptance and completion of the self-management intervention. It is therefore possible that the introduction session and follow-up phone call helped increase acceptability and program engagement in this study. One way to improve eHealth intervention adherence and completion rates could therefore be to increase the level of contact between health care/research study personnel and participants throughout the intervention [65], which is also one reason the research team decided to change from one to two follow-up phone calls for the planned future RCT.

As the primary aim of this study was to examine feasibility, including system use, usability, and ease of use, psychosocial outcome measures were primarily included to test feasibility of use. Potential pre-post effects were only examined as preliminary indications, and findings did not yield relevant results. Despite limited findings, the potential positive impact on HRQoL can be considered promising. Additionally, data variability was large, which may indicate that even though some participants may not have benefited greatly from the EPIO intervention program, others may have benefited greatly. The statistically significant (*P=*.02) finding showing reduced pain acceptance at the 3-month follow-up in this study is challenging to interpret. However, given that almost half (24/50, 48%) of the participants in the study reported having lived with chronic pain for more than 10 years, it may be overly optimistic to expect improvement in pain acceptance, particularly that the “willingness to accept” subscale would be impacted in such a short time. On the other hand, the trend toward reduced pain catastrophizing could be a positive indication. Future research should pay attention to these issues though, preferably also undertaking qualitative interviews to aid in intervention program use, usability, and effect interpretations.

### Study Limitations and Strengths

This study has some limitations. First, the study was designed to assess the feasibility of a digital self-management intervention program in support of patients living with chronic pain; therefore, participants were not randomized, all participants received the intervention, and no definitive statements regarding the effectiveness of the intervention could be made. The study did however successfully establish feasibility and acceptability as intended, with acceptable system use and excellent perceived usefulness and ease of use. Second, the participants were recruited through social media and collaborating partners, and it may be assumed that the study population consisted of highly motivated people. This study therefore cannot conclude whether patients living with chronic pain would in general be interested in or benefit from such an intervention. The strong indications of feasibility in this study, with high acceptability/usability, are however promising. It should also be noted that as the EPIO program is designed to support patients living with chronic pain in general (ie, not pain condition specific), this study was not designed to examine differences in results based on the pain condition. The majority of the participants in this study were Caucasian, women, and those with higher education. People with higher education may be more likely to use health apps in general, while women may be more likely to use self-care or self-management apps and tend to exhibit the highest adherence to digital interventions [68]. Future studies should strive to incorporate user testing and recruitment strategies that may include a wider representation of potential end user groups (eg, gender, education, and ethnicity) in order to further test generalizability.

This study has several strengths. The EPIO intervention is built on clinical and research-based scientific evidence, and is designed and developed in collaboration with end users and related health care personnel, which is a definite strength. The extensive data gathering of 6-month logged system use, as well as usefulness and ease of use examinations are important study strengths. In addition, the inclusion of participants with a variety of pain-related diagnoses can be considered a strength. The goal of the EPIO intervention is to contribute to reducing the negative impact of chronic pain, no matter which type of pain patients experience, and as such, the current feasibility findings may be indicative for patients living with chronic pain in the general population.

### Future Directions

This study established the feasibility of the digital EPIO pain self-management intervention. Suggestions for adjustments needed for optimizing and preparing a future RCT were made, and qualitative interviews for further data exploration were suggested. Additionally, the type of blended care delivery used in this study has the potential to enhance accessibility and actual use of psychosocial interventions and to enhance outreach for patients living with chronic pain. As adherence continuously appears to represent a major challenge for the success of eHealth interventions, future research should explore how aspects of design and development (eg, user involvement and prototype adherence testing) as well as delivery (eg, blended care, continuous follow-up, and social support features) could strengthen intervention adherence in future studies.

Given the mixed findings in the literature related to the utility of eHealth interventions for older adults [66], future research should also aim to incorporate ways to help older adults adopt eHealth interventions. This could potentially be done by including older adults in the design and development process of eHealth interventions [69], but perhaps even more importantly, providing proper training and education for participants on introducing such interventions [69,70]. A future EPIO intervention RCT should also explore this issue further. As system log data examinations revealed continued use and slightly higher program completion rates at the 6-month than 3-month follow-up, future research should examine the preferred and perhaps most likely to be effective period of intervention program use for patients living with chronic pain. Finally, rather than attempting to interpret efficacy outcomes from feasibility findings, efficacy must be evaluated in a future large-scale RCT with long-term follow-up. In the future, examining whether interventions, such as EPIO, have more or less potential for impact based on the type of pain/pain condition targeted is of importance.

### Conclusions

This feasibility pilot study showed how digital self-management intervention programs, such as EPIO, a cognitive-behavioral eHealth pain self-management intervention, may be of use and support for patients living with chronic pain. EPIO program completion rates were acceptable, program feasibility and acceptability were established, and the program was rated as useful and easy to use, with excellent user satisfaction. Intervention program optimization and efficacy testing in a large-scale RCT are warranted and in progress.

## Appendix

Multimedia Appendix 1 A study-specific questionnaire for perceived usefulness and ease of use.

Multimedia Appendix 2 CONSORT-EHEALTH checklist (V 1.6.1).

## Abbreviations

ACT: acceptance and commitment therapy
BPI: Brief Pain Inventory
CBT: cognitive behavioral therapy
CPAQ: Chronic Pain Acceptance Questionnaire
HADS: Hospital Anxiety and Depression Scale
HRQoL: health-related quality of life
MD: mean difference
PCS: Pain Catastrophizing Scale
RCT: randomized controlled trial
SRF: Self-Regulatory Fatigue
SUS: System Usability Scale
TSD: Services for Sensitive Data
