# Supplementary material for: Digital Self-Management in Support of Patients Living With Chronic Pain: Feasibility Pilot Study
Source: JMIR Form Res. 2020 Oct 23;4(10):e23893. doi: 10.2196/23893 (PMC7647816; doi:10.2196/23893)
Supplement: Multimedia Appendix 1 [file formative_v4i10e23893_app1.docx]

# Perceived usefulness and ease of use

## What do you think about using this pain management program?

| **Please mark the answer that best suits you:** | **Totally agree** | **Agree** | **Neither agree nor disagree** | **Disagree** | **Totally disagree** |
| --- | --- | --- | --- | --- | --- |
| 1. The program was easy to use |  |  |  |  |  |
| 1. The exercises were easy to understand |  |  |  |  |  |
| 1. The program was useful |  |  |  |  |  |
| 1. What did you like the best? |  | | | | |
| 1. What did you like the least? |  | | | | |
| 1. Suggestions for improvement? |  | | | | |
